# Mooring Line Damping Estimation for a Floating Wind Turbine

**DOI:** 10.1155/2014/840283

**Published:** 2014-08-27

**Authors:** Dongsheng Qiao, Jinping Ou

**Affiliations:** ^1^Deepwater Engineering Research Center, Dalian University of Technology, Dalian 116024, China; ^2^State Key Laboratory of Coastal and Offshore Engineering, Dalian University of Technology, Dalian 116024, China

## Abstract

The dynamic responses of mooring line serve important functions in the station keeping of a floating wind turbine (FWT). Mooring line damping significantly influences the global motions of a FWT. This study investigates the estimation of mooring line damping on the basis of the National Renewable Energy Laboratory 5 MW offshore wind turbine model that is mounted on the ITI Energy barge. A numerical estimation method is derived from the energy absorption of a mooring line resulting from FWT motion. The method is validated by performing a 1/80 scale model test. Different parameter changes are analyzed for mooring line damping induced by horizontal and vertical motions. These parameters include excitation amplitude, excitation period, and drag coefficient. Results suggest that mooring line damping must be carefully considered in the FWT design.

## 1. Introduction

Renewable wind energy has attracted the attention of many countries, and the development has been from onshore to offshore. Nowadays, the occupied proportion of offshore wind energy is growing in the wind energy industry. As water depth increases, the foundations of offshore wind turbines change from the traditional bottom-mounted substructures to floating support platforms because of economic reasons. The first operational floating offshore wind turbine is Hywind, which works at a water depth of 220 m in the North Sea of Norway [1]. Then, many scholars proposed numerous types of floating wind turbine (FWT), mainly referencing from the offshore oil and gas industry [2–4]. Three primary concepts are classified according to the means of achieving static stability in pitch and roll direction: tension leg platform (TLP), spar buoy, and barge [5]. Some hybrid concepts are also proposed: these concepts include the semi-submersible floating platform system [6] and combined tension leg-mooring line system [7]. These FWT concepts involve the mooring system for station keeping.

Linear frequency domain analysis is commonly employed in the preliminary FWT design, which is the same as that in the offshore oil and gas industry. However, the turbine mass must be added in the global body mass matrix, and the contributions of rotor aerodynamics and gyroscope must be added in the global restoring and damping matrices. Similar studies investigated different types of FWT, such as TLP [8], spar buoy [9], and barge [10]. The mooring system is simplified as a linear spring, and the stiffness is derived from the mean offset of a platform in the restoring properties of the mooring system. Nonlinear dynamic characteristics cannot be considered in the linear frequency domain analysis.

Some scholars studied the global responses of FWT in time domain, in which nonlinear dynamic characteristics can be considered. The key issues include the hydrodynamic loading, the dynamic coupling between the platform and wind turbine motions, and the dynamic coupling between the floating platform and mooring systems. However, most scholars focused on the dynamic coupling between the platform and wind turbine motions. Fulton et al. [11] and Withee [12] applied different coupled models between the platform motion and hydrodynamic loading of TLP designs for 5-MW and 1.5-MW wind turbines in time domain, respectively. Jonkman [13] established a fully coupled time domain aero-hydro-servo-elastic model to analyze the dynamics of FWT, including linear hydrostatic restoring, nonlinear viscous drag, and the added mass and damping contributions from linear wave radiation, and quasistatic mooring line module.

The mooring lines in these models are treated as static or quasistatic module, which ignores the dynamic characteristics of a mooring line. However, the dynamics of a mooring line should not be ignored when the water depth increases, and the reason is that the mooring line damping could significantly affect the motion responses of platform [14, 15].

The mooring line damping for a traditional catenary mooring system results from the line hydrodynamic drag with possible vortex-induced vibration, line internal forces, and line friction on the seabed. Hydrodynamic drag and friction damping are significantly affected by the motion of a floating platform aside from the internal damping caused by material properties. Many studies investigated the contribution of mooring line damping to the global responses of a deepwater floating platform, such as the quasistatic model series of mooring damping calculation [16–19]. Webster [20] used a time domain finite element approach for the parametric study of mooring line damping induced by both horizontal and vertical top end oscillations, and the parameters used in this analysis step from a dimensional analysis. Brown and Mavrakos [21] presented a comparative study on mooring line dynamics and damping based on both time domain and frequency domain methods. Thomas and Hearn [22] indicated that the out-of-plane seabed friction can be negligible and that in-plane effects can influence the peak dynamic tension. Kitney and Brown [23] presented two different scale experiments to validate the results of other scholars. Qiao and Ou [24] calculated the hydrodynamic drag damping of single-component mooring line and obtained linearized damping coefficients.

However, the mooring system design for FWT is different from the deepwater floating platform. The typical installation water depth of FWT is substantially shallow (i.e., <300 m), whereas the deepwater floating platform is larger than 500 m. The top-end dynamics introduced by turbine rotation provides additional strain to the mooring system. Thus, the mooring damping estimation for FWT is necessary to satisfy the requirements for a safe station keeping. The results contribute to the coupled model between the FWT and its mooring system, such as adding the mooring damping in the linear frequency domain analysis.

This study investigates the estimation of mooring line damping on the basis of National Renewable Energy Laboratory 5 MW offshore wind turbine model that is mounted on the ITI Energy barge. A numerical estimation method is derived from the energy absorption of a mooring line resulting from FWT motion. Different parameter changes are analyzed for mooring line damping induced by horizontal and vertical motions. The results of this study could help improve the mooring system design of FWT.

## 2. Numerical Model

### 2.1. Motion Governing Equation of FWT

The FWT is a traditional system with heavy nacelle and rotor on the top, and the whole structural stability finally depends on the mooring system. Therefore, the hydrodynamic analysis coupled with wind loads and mooring system should be considered.

The FWT support platform is simplified as a six rigid-body modes of motion. The equations of motion are as follows:

(1) (Ms+Md)x¨(t)=Fw(t)+Fc(t)+Fsd⁡(t)+Fm(t)+Fh(t)+Fk(t)+Fd(t)+Fp(t),

where $\ddot{x}(t)$ is the acceleration vector; *M*_*s*_ is the matrices of structural mass/inertia; *M*_*d*_ is the added mass/inertia; *F*_*w*_(*t*) is the wind force; *F*_*c*_(*t*) is the current force; *F*_*sd*⁡_(*t*) is the drift force; *F*_*m*_(*t*) is the mooring forces; *F*_*h*_(*t*) is the hydrostatic forces; *F*_*k*_(*t*) is the wave Froude-Krylov force; *F*_*d*_(*t*) is the wave diffraction force; *F*_*p*_(*t*) is the damping force.

**Figure 1:**
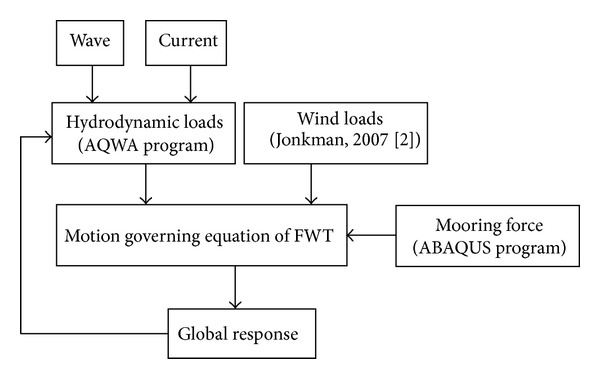
Computational sketch.

The coupled model is calculated by our program in MATLAB, and the simplified computational sketch is shown in Figure 1.

The wave loads are calculated through panel method which is based on potential flow theory, and the software of AQWA Program [25] is used. The current loads are considered as static force, which could be also calculated by the software of AQWA Program. The wind loads mainly depend on the blade aerodynamic characters and the control strategy of the turbine, and the wind loads information is chosen from the research results by Jonkman [2]. The specific equations for calculating wind, wave, and current loads are omitted here, and these could be referenced by Ren [26].

The dynamic mooring forces are considered here, and the specific descriptions are shown in Section 2.2.

### 2.2. Governing Equation of the Mooring Line

The mooring line is generally presumed to be a completely flexible component during motion response analysis. The following motion-governing equation was proposed by Berteaux [27]:

(2) $$\begin{array}{c} m\frac{\partial \vec{V}}{\partial t}={\vec{F}}_{\text{Dn}}+{\vec{F}}_{\text{Dt}}+{\vec{F}}_{\ln}+{\vec{F}}_{\text{lt}}+\frac{\partial \vec{T}}{{\partial s}^{\prime }}+\vec{G} \end{array}$$

(3) $$\begin{array}{c} {\vec{F}}_{\text{Dn}}=\frac{1}{2}\rho_{w}C_{\text{Dn}}D\left|\Delta {\vec{V}}_{n}\right|\Delta {\vec{V}}_{n} \end{array}$$

(4) $$\begin{array}{c} {\vec{F}}_{\text{Dt}}=\frac{1}{2}\rho_{w}C_{\text{Dt}}\pi D\Delta {\vec{V}}_{t}\left|\Delta {\vec{V}}_{t}\right| \end{array}$$

(5) $$\begin{array}{c} {\vec{F}}_{\ln}=\frac{1}{4}\rho_{w}\pi D^{2}C_{\text{mn}}\left(\frac{\partial {\vec{U}}_{n}}{\partial t}-\frac{\partial {\vec{V}}_{n}}{\partial t}\right) \end{array}$$

(6) $$\begin{array}{c} \vec{F_{\text{It}}}=\frac{1}{4}\rho_{w}\pi D^{2}C_{\text{mt}}\left(\frac{\partial {\vec{U}}_{t}}{\partial t}-\frac{\partial {\vec{V}}_{t}}{\partial t}\right), \end{array}$$

where *m* is the mass of the mooring line (per unit length); $\vec{V}$ is the velocity vector of the mooring line; ${\vec{F}}_{\text{Dn}}$ is the normal drag force of the mooring line (per unit length); ${\vec{F}}_{\text{Dt}}$ is the tangential drag force of the mooring line (per unit length); ${\vec{F}}_{\mathrm{In}}$ is the normal inertia force of the mooring line (per unit length); ${\vec{F}}_{\text{It}}$ is the mooring line tangential inertia forces (per unit length). $\partial \vec{T}/\partial s^{\prime }$ is the partial derivative of mooring line tension $\vec{T}$ per arc length of extended mooring line *s*′, and it describes the tension change of a mooring line elementary length *ds*′; $\vec{G}$ is the net weight of the mooring line; *ρ*_*w*_ is the fluid density; *C*_Dn_ is the normal drag coefficient; *D* is the wire diameter; $\Delta {\vec{V}}_{n}$ is the relative normal velocity of the fluid; *C*_Dt_ is the tangential drag coefficient; $\Delta {\vec{V}}_{t}$ is the relative tangential velocity of the fluid; *C*_mn_ is the normal added mass coefficient; ${\vec{U}}_{n}$ is the normal fluid velocity vector at the mooring line direction; ${\vec{V}}_{n}$ is the normal velocity vector of the mooring line; *C*_mt_ is the tangential added mass coefficient; $\vec{U_{t}}$ is the tangential fluid velocity vector at the mooring line direction; and ${\vec{V}}_{t}$ is the tangential velocity vector of the mooring line.

As far as (2) is concerned, the motion-governing equation is a strong complex time-varying nonlinear equation that can be solved using a numerical method. In this paper, the nonlinear finite element program ABAQUS is used for the solution. In ABAQUS, the mooring line is simulated as a hybrid beam element, and the Newton-Raphson iterative method is used to directly solve the nonlinear problem.

### 2.3. Mooring Line Damping

The energy dissipated *E* during one surge oscillation of period *τ* is given by

(7) $$\begin{array}{c} E=\overset{\tau }{\underset{0}{\int }}T_{n}\frac{dq_{n}}{dt}dt, \end{array}$$

where *T*_*n*_ is the component of tension in the direction *n* and *q*_*n*_ is the instantaneous displacement in that direction.

It is often convenient to express this damping in terms of an equivalent linear damping coefficient *B*_*n*_. Then, the instantaneous value of *T*_*n*_ can be given by

(8) $$\begin{array}{c} T_{n}=B_{n}\frac{dq_{n}}{dt}. \end{array}$$

If the oscillation is sinusoidal with an amplitude, *q*_0_, and a period, *τ*, the approximate absorbed energy *E* is given by

(9) $$\begin{array}{c} E=\overset{\tau }{\underset{0}{\int }}T_{n}\frac{dq_{n}}{dt}\;dt=B_{n}{\overset{\tau }{\underset{0}{\int }}\left[\frac{dq_{n}}{dt}\right]}^{2}dt=\frac{2\pi^{2}q_{0}^{2}B_{n}}{\tau }. \end{array}$$

Consequently, the energy dissipated by the mooring line during one surge oscillation can be computed, and then the linear damping coefficient is obtained by

(10) $$\begin{array}{c} B_{n}=\frac{E\tau }{2\pi^{2}q_{0}^{2}}. \end{array}$$

The dissipated energy *E* can be obtained by integrating the work done by the upper tension during one surge oscillation.

Based on the nonlinear finite element dynamic analysis shown in Section 2.2, the mooring damping is calculated by our program in MATLAB.

## 3. Experiment and Results

The validation of motion governing equation of FWT in Section 2.1 has been conducted by Ren [26]. In his research, the wind and wave loads are considered, and the quasistatic mooring line module is used. Due to the fact that the main aim of the analysis in this paper is to estimate the mooring damping of FWT, the following experiment is designed, which is only used to validate the numerical calculation of dynamic mooring forces.

### 3.1. Experimental Setup

**Figure 2:**
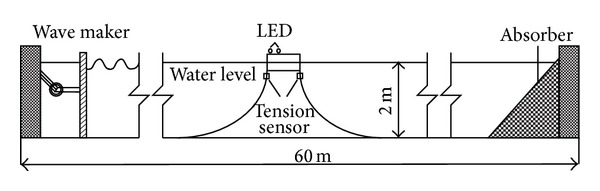
A sketch of model setup in wave tank.

A prototype system consisting of a cubic floater and two symmetrically arranged lines in 160 m water depth is model tested in the Nonlinear Wave Tank, State Key Laboratory of Coastal and Offshore Engineering, Dalian University of Technology, to validate the numerical simulation method used in this study. The wave tank has a 60 m length, a 4 m width, and a 2 m operating water depth. The model scale is 1/80 by considering the water depth of the tank and the capability of the wave maker and other instruments. A schematic of the wave tank and the total experimental system configuration used in the present investigations are shown in Figure 2.

The floater model has a 50 cm length, a 50 cm width, a 20 cm height, and a 2 cm thickness. The height of the gravity center is 6 cm as referred to the underside of the box, and the draft is also 6 cm. Two mooring lines are symmetrically arranged at both sides under the box (Figure 2). The top end of the mooring line is connected to the underside of the box through a tension sensor, whereas the other end is fixed at the bottom of the tank with a horizontal span of 5.75 m. The mooring line is modeled by a wire rope, and the main properties are shown in Table 1.

Tension sensors are connected at the top between the lines and floater to measure the tensions acting on the mooring lines. The customized FQ-1 type tension sensors, each of 2.0 kg capacity with a comprehensive accuracy of 0.03% FS, are equipped with a digital signal recorder box for data acquisition. Three degrees of freedom motions (surge, heave, and pitch) of the floater are measured by a CCD camera system that uses a high-speed camera sensing two LED signals (Figure 2) attached on the model.

The system was tested at selected regular wave excitation frequencies. The wave period was varied from 14 s to 35 s, and the wave height was 6 m, all referred to the prototype scale because of the limited capacity of the wave generator in the laboratory.

### 3.2. Experimental Results

**Figure 3:**
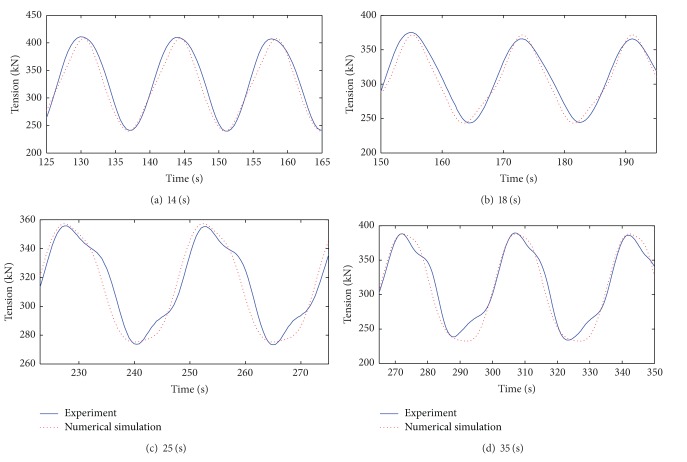
Tension time series.

**Figure 4:**
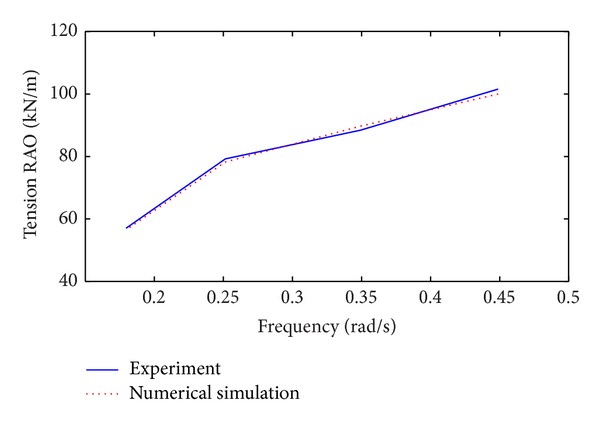
RAO of the mooring line tension.

The tension time series of mooring lines were obtained on the basis of numerical simulation. The platform was tested at selected regular wave excitation frequencies, and the tension time series of the numerical simulation and experimental mooring lines were compared (Figure 3). The response amplitude ratio (RAO) of the line tension to the fairlead motion is shown in Figure 4. The RAO is defined as the mean double amplitude of the mooring line tension to the mean double amplitude of the fairlead motion.

The results shown in Figures 3 and 4 validate the numerical simulation. The time series of the simulated and experimental mooring line tensions agree, and the period is equal to the wave excitation period. The RAOs of the mooring tension in the numerical simulation and in the experimental results also agree. These results suggest that the numerical simulation method can be directly used in the following study.

## 4. Mooring System of FWT

### 4.1. Mooring System Layout

The National Renewable Energy Laboratory 5 MW offshore wind turbine is used as the basic model, and details on the properties of the model are provided in [28]. The ITI Energy barge, which was developed by the Department of Naval Architecture and Marine Engineering at the Universities of Glasgow and Strathclyde with ITI Energy [10], is used for the support structure. The main properties of the ITI Energy barge are listed in Table 2.

**Figure 5:**
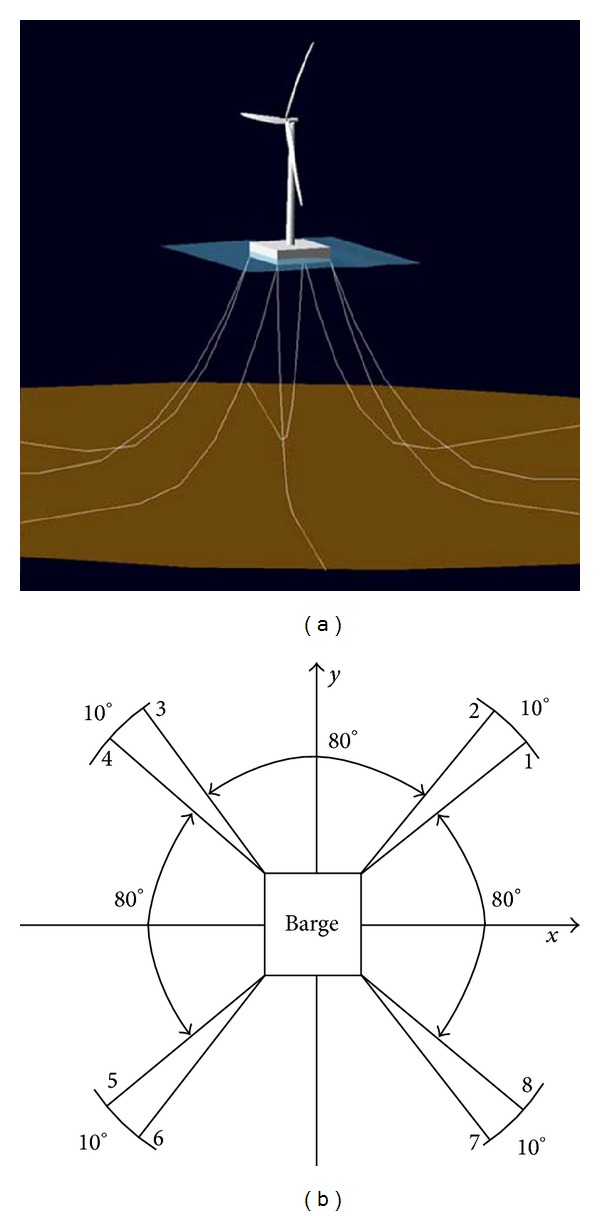
Mooring system configuration of FWT. (a) ITI Energy barge. (b) Mooring system layout.

The mooring system consists of four (2 × 4) groups (Figure 5). The groups in the mooring system are positioned 90° apart, and the lines in each group are positioned 10° apart.

### 4.2. Baseline of the Mooring System

**Figure 6:**
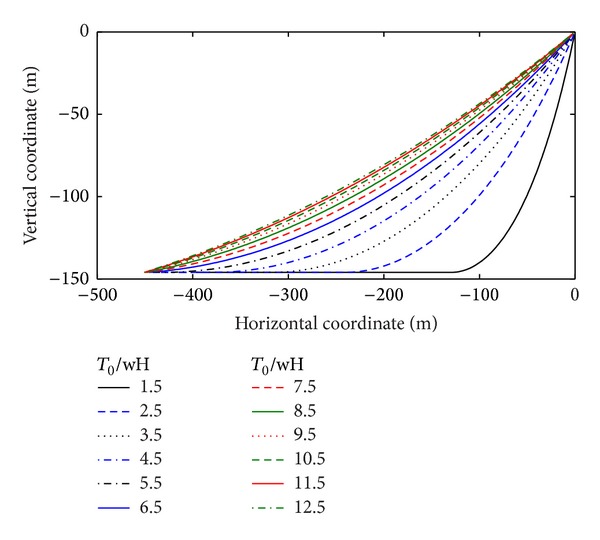
Mooring line geometry with nondimensional tension.

The number 1 mooring line is used as the baseline in this study. The different pretensions (*T*_0_) are performed in the numerical simulation to investigate the behavior of the baseline. In the nondimensional analysis, *T*_0_/*wH* represents the nondimensional pretension, and *E*/*q*_*o*_ *wH* represents the nondimensional damping of the mooring line. Here, *w* is the weight per unit length of the line in water, *H* is the water depth, *B*_*n*_ is the mooring damping, and *q*_*o*_ is the motions amplitude.  Figure 6 shows a profile view of the baseline under different pretensions.

## 5. Parametric Variations

The influences of different parametric variations on the mooring line damping are investigated according to the numerical simulation method mentioned above. The parameters include excitation amplitude (*q*_0_), excitation period (*τ*), and drag coefficient (*C*_*d*_).

The nondimensional *q*_0_/*H* and $\left(\tau /2\pi \right)\sqrt{g/H}$ are selected in horizontal and vertical motions on the basis of the motion responses analysis of ITI Energy barge and the nondimensional analysis, respectively. Only one parameter changes at a time while the other two parameters are kept unchanged.

For the baseline (number 1), the initial nondimensional parameters are *T*_0_/*wH* = 2.5, *q*_0_/*H* = 0.03 (horizontal motions), *q*_0_/*H* = 0.008 (vertical motions), $\left(\tau /2\pi \right)\sqrt{g/H}=2.4$ (horizontal motions), $\left(\tau /2\pi \right)\sqrt{g/H}=0.4$ (vertical motions), and *C*_*d*_ = 1.2.

### 5.1. Effects of Excitation Amplitude

**Figure 7:**
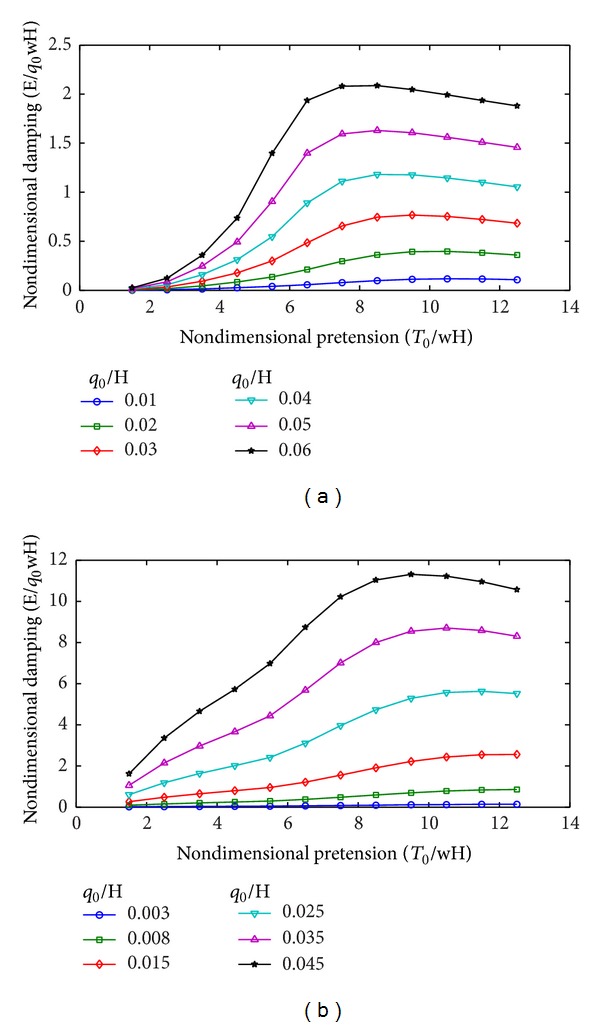
Variation of mooring line damping with excitation amplitude. (a) Horizontal motions. (b) Vertical motions.

Table 3 and Figure 7 show the effects of excitation amplitude on mooring damping when the other parameters remain unchanged. The characters of the curves are similar in the horizontal and vertical motions. The nondimensional damping initially increases and then decreases with increasing nondimensional pretension. The nondimensional damping increases with increasing excitation amplitude.

For the horizontal motions, the damping reaches a peak as the nondimensional pretension approaches 8, and the peak achieved for small amplitudes is slightly larger than that for large amplitudes. For the vertical motions, the damping reaches a peak when the non-dimensional pretension approaches 10, and the peak achieved for small amplitudes is slightly larger than that for large amplitudes.

The peak non-dimensional damping of the horizontal motions is only approximately 10% that of the vertical motions. The reason is that, for the baseline, the mooring damping under the horizontal and vertical excitations is mainly focused on the low and wave frequency ranges, respectively. This phenomenon indicates that the mooring line damping in the wave frequency range should also be considered in the vertical motion prediction of the platform.

For small pretension, the non-dimensional damping (for both horizontal and vertical motions) increases as the non-dimensional pretension increases. This result corresponds to a nearly constant equivalent linear damping coefficient. For large pretension, the non-dimensional damping (for both horizontal and vertical motions) becomes less dependent on the nondimensional pretension. The reason is that the mooring line geometry changes from slack to taut with increasing pretension, and the mooring line length affects the damping contribution. When the nondimensional pretension reaches 6.5, the total mooring line is lifted without lying segment. Thus, the damping becomes less dependent on the nondimensional pretension for large pretension.

### 5.2. Effects of Excitation Period

**Figure 8:**
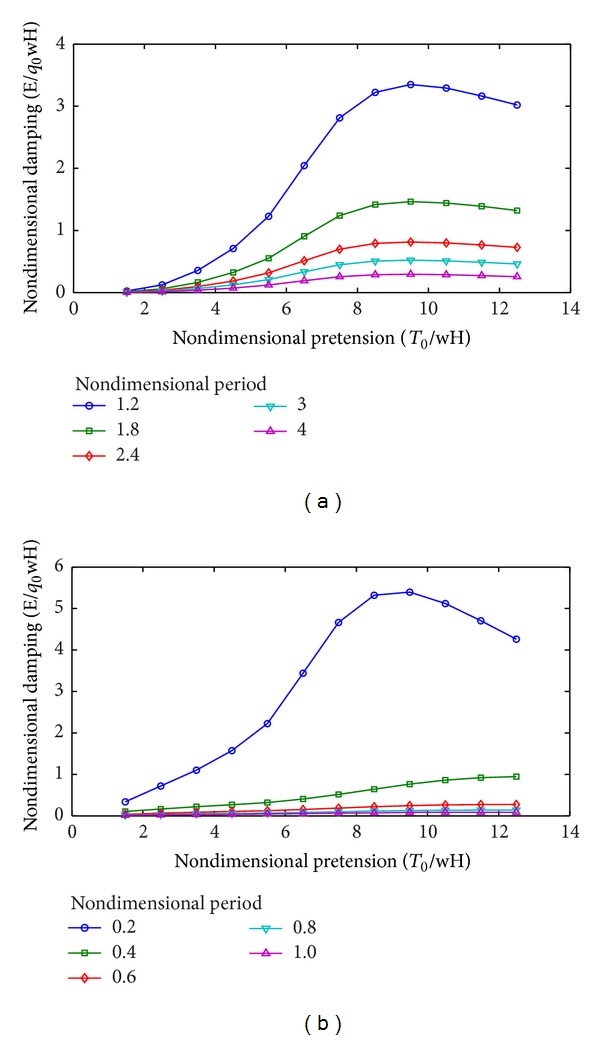
Variation of mooring line damping with excitation period. (a) Horizontal motions. (b) Vertical motions.

Table 4 and Figure 8 show the effects of excitation period on mooring damping when the other parameters remain unchanged.

The nondimensional damping decreases for both the horizontal and vertical motions as the excitation period increases. The reason is that the dynamic tension becomes larger than the quasistatic tension under the high frequency (short period) excitation. Meanwhile, the mooring line damping increases. When the nondimensional period increases twice, the nondimensional damping becomes approximately 1/4 of that for all nondimensional pretensions (both horizontal and vertical motions).

When the nondimensional period is approximately 1.0, the nondimensional damping for the horizontal excitation is approximately 30 times that for the vertical excitation. For the same value of non-dimensional damping, the corresponding non-dimensional period for the horizontal excitation is in the low frequency range, while the corresponding non-dimensional period for the vertical excitation is in the wave frequency range. This phenomenon further indicates that the mooring line damping in the wave frequency range should be considered in the vertical motion prediction of the platform.

### 5.3. Effects of Drag Coefficient

**Figure 9:**
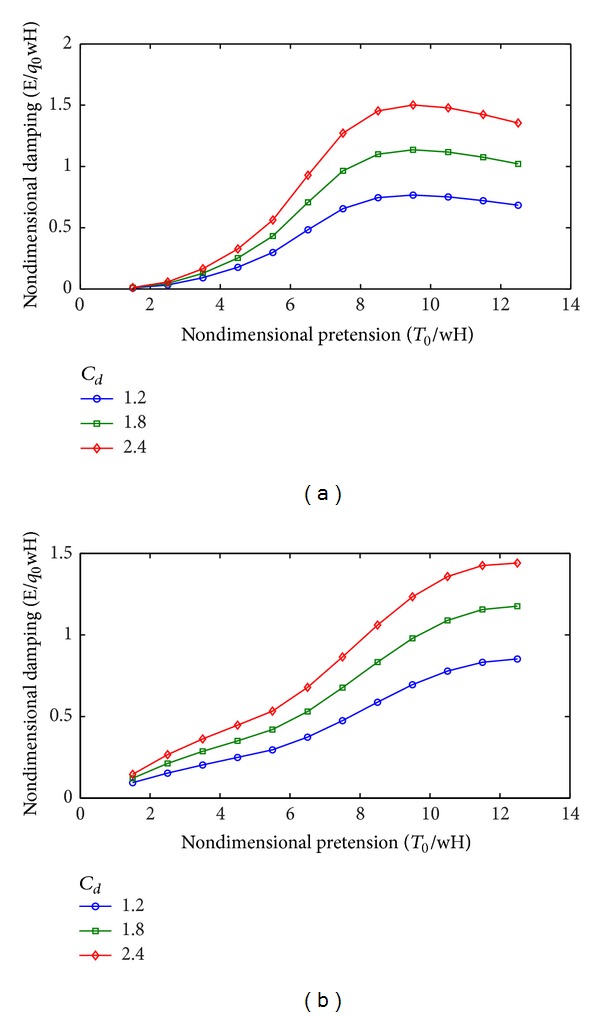
Variation of mooring line damping with drag coefficient. (a) Horizontal motions. (b) Vertical motions.

Table 5 and Figure 9 show the effects of drag coefficient on mooring damping when the other parameters remain unchanged. The characters of the curves show some differences in the horizontal and vertical motions. For the horizontal motions, the nondimensional damping initially increases and then decreases with increasing nondimensional pretension. The damping reaches a peak when the nondimensional pretension reaches 9.5. For the vertical motions, the nondimensional damping increases as the nondimensional pretension increases and reaches 12.5 in this paper.

With increasing drag coefficient, the nondimensional damping almost linearly increases. This phenomenon indicates that the drag coefficient should be prudently selected in the design.

## 6. Conclusions

The mooring line damping estimation is studied on the basis of the National Renewable Energy Laboratory 5 MW offshore wind turbine model that is mounted on the ITI Energy barge. The numerical estimation method is derived from the energy absorption by a mooring line resulting from the FWT motion. The numerical method is validated by a 1/80 scale model test. The following preliminary findings are obtained.

1. The nondimensional damping initially increases and then decreases with increasing nondimensional pretension.
2. The nondimensional damping increases with increasing excitation amplitude. The peak nondimensional damping of the horizontal motions is only approximately 10% that for the vertical motions. The mooring line damping in the wave frequency range should be considered in the vertical motion prediction of the platform.
3. For small pretension, the nondimensional damping increases with increasing nondimensional pretension. For large pretension, the nondimensional damping becomes less dependent on the nondimensional pretension.
4. The nondimensional damping decreases for both the horizontal and vertical motions with increasing excitation period. When the nondimensional period increases twice, the nondimensional damping becomes approximately 1/4 of that for all nondimensional pretensions.
5. With increasing drag coefficient, the nondimensional damping almost linearly increases.

The mooring line damping is clearly a complex phenomenon that shows some differences in the horizontal and vertical excitations. The estimation results for the mooring damping of a FWT can help in the motion prediction and mooring design of the FWT.

## Figures and Tables

**Table 1:** Physical properties of mooring line.

Item	Prototype	Experiment
Length (m)	500	6.25
Diameter (mm)	160	2
Submerged weight per unit length (N/m)	778	0.122
Axial stiffness (N)	1.5*E*9	2930

**Table 2:** Properties of ITI Energy barge.

Item	Value
Width × length (m)	40 × 40
Draft (m)	4
Water displacement (m^3^)	6000
Mass, including ballast (kg)	5452000
CM location below still water level (m)	0.2818
Roll inertia about CM (kg*·*m^2^)	726900000
Pitch inertia about CM (kg*·*m^2^)	726900000
Yaw inertia about CM (kg*·*m^2^)	1454000000
Number of mooring lines	8
Depth to fairleads, anchors (m)	4, 150
Radius to fairleads, anchors (m)	28.28, 423.4
Unstretched line length (m)	473.3
Line diameter (m)	0.0809
Line mass density (kg/m)	130.4
Line extensional stiffness (N)	589000000

**Table 3:** Mooring line damping estimation under different excitation amplitude.

Nondimensional tension (*T*_0_/*wH*)	Nondimensional damping (*E*/*q*_*o*_ *wH*)											
	Horizontal motion (*q*_0_/*H*)						Vertical motion (*q*_0_/*H*)					
	0.01	0.02	0.03	0.04	0.05	0.06	0.003	0.008	0.015	0.025	0.035	0.045
1.5	0.002	0.004	0.008	0.012	0.018	0.025	0.019	0.095	0.266	0.606	1.053	1.618
2.5	0.006	0.016	0.033	0.057	0.086	0.123	0.027	0.153	0.477	1.184	2.144	3.354
3.5	0.014	0.044	0.092	0.158	0.246	0.358	0.035	0.204	0.648	1.632	2.970	4.659
4.5	0.025	0.084	0.177	0.311	0.492	0.736	0.043	0.249	0.796	2.010	3.662	5.729
5.5	0.039	0.135	0.299	0.546	0.905	1.396	0.050	0.296	0.951	2.415	4.434	6.978
6.5	0.057	0.211	0.484	0.891	1.398	1.935	0.062	0.374	1.213	3.109	5.682	8.742
7.5	0.078	0.296	0.656	1.111	1.594	2.080	0.078	0.475	1.553	3.962	7.005	10.221
8.5	0.098	0.360	0.745	1.180	1.629	2.086	0.095	0.588	1.910	4.733	7.998	11.041
9.5	0.111	0.392	0.767	1.177	1.606	2.046	0.112	0.695	2.220	5.285	8.549	11.311
10.5	0.117	0.396	0.752	1.145	1.561	1.992	0.125	0.779	2.435	5.570	8.706	11.228
11.5	0.115	0.382	0.721	1.102	1.510	1.935	0.135	0.832	2.546	5.628	8.587	10.957
12.5	0.108	0.359	0.684	1.054	1.457	1.879	0.139	0.853	2.560	5.520	8.306	10.575

**Table 4:** Mooring line damping estimation under different excitation period.

Nondimensional tension (*T*_0_/*wH*)	Nondimensional damping (*E*/*q*_*o*_ *wH*)									
	Horizontal period $\left(\left(\tau /2\pi \right)\sqrt{g/H}\right)$					Vertical period $\left(\left(\tau /2\pi \right)\sqrt{g/H}\right)$				
	1.2	1.8	2.4	3	4	0.2	0.4	0.6	0.8	1.0
1.5	0.027	0.013	0.008	0.005	0.003	0.339	0.104	0.036	0.020	0.014
2.5	0.126	0.059	0.035	0.024	0.015	0.723	0.165	0.066	0.037	0.024
3.5	0.356	0.165	0.097	0.065	0.039	1.103	0.220	0.088	0.049	0.033
4.5	0.709	0.324	0.188	0.125	0.074	1.571	0.270	0.106	0.059	0.039
5.5	1.228	0.552	0.317	0.208	0.122	2.227	0.322	0.123	0.068	0.045
6.5	2.044	0.907	0.513	0.334	0.192	3.441	0.406	0.152	0.083	0.054
7.5	2.813	1.240	0.697	0.450	0.257	4.665	0.518	0.186	0.100	0.064
8.5	3.223	1.417	0.792	0.509	0.289	5.321	0.644	0.220	0.116	0.074
9.5	3.349	1.464	0.815	0.522	0.294	5.396	0.765	0.246	0.129	0.082
10.5	3.291	1.442	0.800	0.511	0.287	5.120	0.860	0.265	0.137	0.086
11.5	3.164	1.389	0.768	0.488	0.273	4.704	0.921	0.273	0.140	0.087
12.5	3.019	1.321	0.728	0.462	0.257	4.259	0.947	0.273	0.138	0.086

**Table 5:** Mooring line damping estimation under different drag coefficient.

Nondimensional tension (*T*_0_/*wH*)	Nondimensional damping (*E*/*q*_*o*_ *wH*)					
	Horizontal motion (*C*_*d*_)			Vertical motion (*C*_*d*_)		
	*C*_*d*_ = 1.2	*C*_*d*_ = 1.8	*C*_*d*_ = 2.4	*C*_*d*_ = 1.2	*C*_*d*_ = 1.8	*C*_*d*_ = 2.4
1.5	0.008	0.010	0.012	0.095	0.122	0.146
2.5	0.033	0.046	0.058	0.153	0.212	0.267
3.5	0.092	0.128	0.165	0.204	0.286	0.363
4.5	0.177	0.252	0.326	0.249	0.351	0.447
5.5	0.299	0.431	0.563	0.296	0.420	0.533
6.5	0.484	0.707	0.929	0.374	0.531	0.679
7.5	0.656	0.964	1.271	0.475	0.676	0.865
8.5	0.745	1.100	1.453	0.588	0.833	1.060
9.5	0.767	1.136	1.501	0.695	0.979	1.233
10.5	0.752	1.117	1.478	0.779	1.089	1.358
11.5	0.721	1.075	1.423	0.832	1.155	1.426
12.5	0.684	1.022	1.355	0.853	1.176	1.441
